# Fast and accurate influenza forecasting in the United States with Inferno

**DOI:** 10.1371/journal.pcbi.1008651

**Published:** 2022-01-31

**Authors:** Dave Osthus

**Affiliations:** Statistical Sciences Group, Los Alamos National Laboratory, Los Alamos, New Mexico, United States of America; London School of Hygiene & Tropical Medicine, UNITED KINGDOM

## Abstract

Infectious disease forecasting is an emerging field and has the potential to improve public health through anticipatory resource allocation, situational awareness, and mitigation planning. By way of exploring and operationalizing disease forecasting, the U.S. Centers for Disease Control and Prevention (CDC) has hosted FluSight since the 2013/14 flu season, an annual flu forecasting challenge. Since FluSight’s onset, forecasters have developed and improved forecasting models in an effort to provide more timely, reliable, and accurate information about the likely progression of the outbreak. While improving the predictive performance of these forecasting models is often the primary objective, it is also important for a forecasting model to run quickly, facilitating further model development and improvement while providing flexibility when deployed in a real-time setting. In this vein I introduce Inferno, a fast and accurate flu forecasting model inspired by Dante, the top performing model in the 2018/19 FluSight challenge. When pseudoprospectively compared to all models that participated in FluSight 2018/19, Inferno would have placed 2nd in the national and regional challenge as well as the state challenge, behind only Dante. Inferno, however, runs in minutes and is trivially parallelizable, while Dante takes hours to run, representing a significant operational improvement with minimal impact to performance. Forecasting challenges like FluSight should continue to monitor and evaluate how they can be modified and expanded to incentivize the development of forecasting models that benefit public health.

## Author summary

Infectious disease forecasting, if accurate, timely, and reliable, can assist decision makers with resource allocation planning in an attempt to curb the negative impacts of an outbreak. Forecasting challenges, like the U.S. Centers for Disease Control and Prevention’s flu forecasting challenge, FluSight, provide a space for teams to develop and operationalize real-time forecasting models that benefit public health, with weekly forecasts made at the state-level, Health and Human Services region-level, and the United States. The ultimate goal of these models is to produce accurate forecasts within the constraints of the forecasting challenge. Having a forecasting model that runs quickly is also important for future scalability, model development, and operational flexibility. In this paper, I present a fast and accurate flu forecasting model, Inferno. Through retrospective comparisons with FluSight-participating models, Inferno was shown to be a leading forecasting model in the field. Inferno, however, runs in minutes not hours, as other leading forecasting models do. This reduction in runtime constitutes an advancement in flu forecasting, positioning Inferno to scale to more granular geographic units, like counties or health care providers.

## 1 Introduction

Infectious disease outbreaks can be disruptive, deadly, and complex. By the end of July 2021, COVID-19 had killed more than 4 million people globally and over 600 thousand people in the United States (U.S.) [1]. Each year in the U.S., seasonal influenza kills tens of thousands of people and hospitalizes hundreds of thousands [2]. Life saving resources, such as respirators, antivirals, vaccines, and medical professionals must be allocated to ensure locations are prepared and ready for the impending outbreak. If infectious disease forecasts can be done accurately and reliably with adequate lead times, they could be used to help inform resource allocation planning.

Infectious disease forecasting is still relatively young, but can no longer claim novelty. There has been a flurry of infectious disease forecasting challenges/collaborations in the last ten years, including the Defense Advanced Research Projects Agency’s 2014/15 Chikungunya challenge [3], a collection of challenges hosted by the U.S. Centers for Disease Control and Prevention (CDC) related to vector-borne diseases such as dengue (2015) [4] and West Nile virus (2020) [5], a challenge predicting the presence of mosquito vectors (2019) [6], the U.S. CDC COVID-19 forecasting collaboration (2020) [7], and the U.S. CDC’s flagship influenza forecasting challenge, FluSight, held annually since the 2013/14 flu season [8–10]. The FluSight challenge alone has resulted in a wave of infectious disease forecasting model development, including mechanistic models [11–13], statistical/machine learning models [14–18], fusions of mechanistic and statistical models [19–21], ensemble models [22], and agent-based models [23].

One necessary ingredient of a forecasting challenge is measured data. As a measure of flu activity, FluSight uses data on influenza-like illness (ILI). ILI is defined as a temperature greater than or equal to 100 degrees Fahrenheit, a cough and/or sore throat, and no other known cause except influenza. Approximately 3,000 outpatient healthcare providers report two pieces of information to the CDC’s U.S. Outpatient Influenza-like Illness Surveillance Network (ILINet) each week: the number of patients seen for any reason and the number of those patients that have an influenza-like illness. These data are then aggregated to the levels of states, Health and Human Services (HHS) regions, and the United States. ILI for a region and week is computed as the number of patients with ILI divided by the number of patients seen for any reason, expressed as a percentage (thus, ILI is between 0% and 100%). In addition to ILI, weighted ILI (wILI) is also computed for HHS regions and the United States. Weighted ILI is computed as a U.S. Census population-weighted average of state ILI estimates and is also between 0% and 100%.

The organizing body of a forecasting challenge (in the case of FluSight, the U.S. CDC) provides immense operational and research value by determining forecasting targets of public health relevance on behalf of and in collaboration with local, state, and national stakeholders (often including the organizing body’s own interests), identifying relevant data sources and making them publicly available to forecasters, and defining the forecast evaluation criteria—a more challenging task than it may first appear (see [24] and [25]).

For instance, for states, HHS regions, and the United States, the FluSight challenge asks forecasters to predict seven targets on a weekly basis throughout the flu season: 1 through 4-week-ahead forecasts of ILI for states or wILI for HHS regions and the U.S. (collectively referred to as (w)ILI), the week of flu season onset, the week the flu season will peak, and the peak value of (w)ILI for the flu season. FluSight uses a modified log scoring rule to evaluate forecasts [26]. The modified log scoring rule evaluates probabilistic forecasts, requiring forecasters to not only provide a prediction of what they think will happen in the future but also quantify how sure they are of that. The choice made by the U.S. CDC to use a modified log scoring rule makes clear their position that uncertainty quantification is of value to public health. Given a set of forecasting targets and an evaluation metric, forecasters participating in FluSight develop models capable of forecasting those targets within the real-time operational constraints of the challenge with the goal of maximizing their model’s forecast evaluation score.

Forecasting challenges are powerful incentivization engines. How they are structured encourage/require models to have certain properties that align with public health needs. For instance, if public health needs forecasting models capable of short-term and long-term forecasting, selecting short-term and long-term/seasonal targets incentives the development of models that can do both of those things well. If public health needs probabilistic forecasting models that quantify their uncertainty, selecting a scoring rule that rewards appropriate uncertainties and penalizes overly confident/conservative forecasts incentivizes probabilistic model development. If public health needs forecasting models to support rapid response decision making, increasing the forecast submission cadence (e.g., from weekly to daily), reducing the amount of time between the release of new data and the forecast submission deadline, and/or augmenting the scope of forecasting geographies (e.g., from HHS regions to states to counties) incentivizes the development of forecasting models that run quickly.

In this paper, I focus on improving the runtime of flu forecasting models while maintaining high prediction standards with the presentation of Inferno, a fast and accurate flu forecasting model. Inferno is a parallelizable, Bayesian forecasting model inspired by Dante, the top performing model in FluSight 2018/19 [14]. The achieved goal of Inferno is to maintain the high predictive performance of Dante but substantially decrease the runtime. As will be discussed later, in a pseudoprospective comparison, Inferno would have placed 2nd only to Dante in the 2018/19 FluSight challenge but runs in minutes rather than hours, constituting a significant speed-up in operational performance.

In the remainder of this paper, I describe the details to Inferno (Section 2) and present Inferno’s forecasting performance as compared to all participating models in FluSight 2018/19 (Section 3).

## 2 Methods

### 2.1 Dante background

Dante is a multiscale, probabilistic, influenza forecasting model. It requires historical data of past flu seasons to effectively learn patterns and leverage those patterns for forecasting. Dante has two sub-models: a state forecasting model and an aggregation model which combines state forecasts to produce forecasts for HHS regions and the United States.

Dante’s state forecasting model is

$$\begin{array}{cccc} y_{rst}|\theta_{rst},\lambda_{r} & ∼\text{Beta}(\lambda_{r}\theta_{rst},\lambda_{r}\left(1-\theta_{rst}\right)) & & \left(\text{Eq}\;\text{1}\;\text{of}\;[14]\right)\\ \theta_{rst} & ={\text{logit}}^{-1}\left(\pi_{rst}\right) & & \left(\text{Eq}\;\text{4}\;\text{of}\;[14]\right)\\ \pi_{rst} & =\mu_{t}^{\text{all}}+\mu_{rt}^{\text{state}}+\mu_{st}^{\text{season}}+\mu_{rst}^{\text{interaction}} & & (\text{Eq}\;\text{5}\;\text{of}\;[14]), \end{array}$$

where *y*_*rst*_ is ILI/100 for week *t* for state *r* during season *s* and *θ*_*rst*_, the conditional expectation of *y*_*rst*_ given *θ*_*rst*_ and λ_*r*_, is modeled as a function of four components: an overall trend component ($\mu_{t}^{\text{all}}$), a state-specific deviation component ($\mu_{rt}^{\text{state}}$), a season-specific deviation component ($\mu_{st}^{\text{season}}$), and a state and season-specific deviation component ($\mu_{rst}^{\text{interaction}}$). These four components are each modeled as random or reverse-random walks—flexible time series models that capture temporal correlation (for more details and non-infectious disease applications of reverse-random walks, see [27] and [28]). By modeling all states and past flu seasons jointly, Dante is able to borrow information across seasons and space. By modeling the HHS regional and United States forecasts as U.S. Census population-weighted averages of state forecasts, Dante ensures self-consistency across geographic scales. For more details on Dante, see [14].

Dante is a fully Bayesian model, capturing uncertainty in all model parameters, latent states, and forecasts through its posterior (predictive) distribution. The fully Bayesian formulation and self-consistency of Dante comes at a computational price, however. Dante represents a large model that will grow each year as more historical data are added and is not well-positioned to scale with possible future changes/expansions to FluSight (e.g., county-level forecasting). Nothing is precomputed and due to its interconnected model structure, it is not obvious how to break up Dante to exploit parallelization.

Inferno was developed to addresses these computational shortcomings. Inferno, while motivated by Dante, deviates from Dante in two main ways. First, Inferno is fit separately to each geographical unit. This allows Inferno to leverage parallel computing architectures but at the expense of modeling correlations across states. Second, Inferno precomputes many of its parameters via a heuristic estimation procedure, reducing the number of parameters and latent model components that need to be sampled via Markov chain Monte Carlo (MCMC). These two choices result in significant computational speed ups with only moderate loss in forecast accuracy. In Section 2.2, I describe the Inferno forecasting model.

### 2.2 Inferno

Inferno is fit to each geographical unit separately and can be viewed as a simplified version of Dante, where Dante’s state-specific components ($\mu_{rt}^{\text{state}}$ and $\mu_{rst}^{\text{interaction}}$) are removed, certain parameters are kept fixed at predetermined values, and the random walk model on $\mu_{st}^{\text{season}}$ is replaced with a multivariate normal model. Specifically, let *y*_*s*,*t*_ ∈ (0, 1) be ILI/100 for states or wILI/100 for HHS regions and the United States for season *s* = 1, 2, …, *S* and week *t* = 1, 2, …, *T* = 35, where *t* = 1 corresponds to Morbidity and Mortality Weekly Report (MMWR) week 40, roughly the beginning of October, and *T* = 35 roughly corresponds to the end of May. Inferno’s generative model is defined as follows, with all parameters which are not assigned a prior distribution set to fixed values (e.g., *γ*_*t*_, $\sigma_{\Sigma }^{2}$; see below):

$$y_{s,t}|\theta_{s,t},\alpha ∼\text{Beta}(\alpha \theta_{s,t},\alpha \left(1-\theta_{s,t}\right))$$ (1)

$$\theta_{s,t}={\text{logit}}^{-1}\left(\gamma_{t}+\delta_{s,t}\right)$$ (2)

$$\delta_{s}|\mu_{s},\Sigma ∼\text{MVN}(\mu_{s}1,\Sigma )$$ (3)

$$\mu_{s}|\sigma_{\mu }^{2}∼\text{N}(0,\sigma_{\mu }^{2})$$ (4)

$$\Sigma_{t,t}=\sigma_{\Sigma }^{2}$$ (5)

$$\Sigma_{t,t^{\prime }\neq t}=\phi \sigma_{\Sigma }^{2}\text{exp}\left(-\lambda {\left(t-t^{\prime }\right)}^{2}\right),$$ (6)

where

- *y*_*s*,*t*_ is the noisy, observable measurement of (w)ILI/100 on week *t* of season *s*.
- *θ*_*s*,*t*_ models the true but unobservable value of (w)ILI/100 on week *t* of season *s*.
- The scalar hyperparameter *α* > 0 helps characterize the variance of *y*_*s*,*t*_|*θ*_*s*,*t*_, *α*.
- *γ*_*t*_ models the typical (w)ILI/100 value on week *t* on the logit scale.
- ***δ***_*s*_ = (*δ*_*s*,1_, *δ*_*s*,2_, …, *δ*_*s*,*T*_)′ is a *T* × 1 vector, where *δ*_*s*,*t*_ models the deviation from the typical (w)ILI/100 value *γ*_*t*_ on the logit scale on week *t* of season *s*.
- ***δ***_*s*_ is modeled with a multivariate normal (MVN) distribution with mean *μ*_*s*_**1** (a scalar *μ*_*s*_ times a *T* × 1 vector of ones) and covariance matrix **Σ**.
- *μ*_*s*_ is the average deviation of ***δ***_*s*_ from ***γ*** = (*γ*_1_, *γ*_2_, …, *γ*_*T*_)′, where $\sigma_{\mu }^{2}>0$ characterizes the season-to-season variability in *μ*_*s*_.
-
$\Sigma_{t,t}=\sigma_{\Sigma }^{2}$ is the variance of *δ*_*s*,*t*_.
-
$\Sigma_{t,t^{\prime }\neq t}=\phi \sigma_{\Sigma }^{2}\text{exp}\left(-\lambda {\left(t-t^{\prime }\right)}^{2}\right)$ is the covariance between *δ*_*s*,*t*_ and *δ*_*s*,*t*′_. The covariance between *δ*_*s*,*t*_ and *δ*_*s*,*t*′_ gets closer to zero as |*t* − *t*′| gets larger.
- The correlation between *δ*_*s*,*t*_ and *δ*_*s*,*t*+1_ is *ϕ*exp(-λ). The hyperparameters λ > 0 and *ϕ* ∈ [0, 1] control the correlation structure, where the correlation between *δ*_*s*,*t*_ and *δ*_*s*,*t*+1_ tends towards *ϕ* as λ approaches 0 and *ϕ* is the upper bound on the correlation (i.e., *ϕ*exp(-λ) < *ϕ* for all λ > 0).

In this paper, bold quantities represent vectors or matrices, while non-bold quantities represent scalars. Because Inferno is applied to each geographical unit *r* separately, the subscript *r* is suppressed throughout. The Beta distribution of Eq 1 requires *y*_*s*,*t*_ ∈ (0, 1). There is no guarantee (w)ILI/100 is not equal to 0 or 1. Thus, all *y*_*s*,*t*_ below a low threshold *l* are set equal to *l* and all *y*_*s*,*t*_ above 1 − *l* are set to 1 − *l*. For this work, *l* = 0.0005 and *y*_*s*,*t*_ is thresholded by *l* for all observations before the modeling begins.

The parameters kept fixed in the above generative model (*α*, ***γ*** = (*γ*_1_, *γ*_2_, …, *γ*_*T*_)′, $\sigma_{\mu }^{2}$, $\sigma_{\Sigma }^{2}$, λ, and *ϕ*) are estimated from past season’s (w)ILI data with a heuristic estimation procedure (at least two past seasons are required to heuristically estimate all Inferno parameters). As will be shown, this heuristic estimation procedure works well in practice to produce forecasts—Inferno’s primary goal—as Inferno’s forecast performance is competitive with Dante. While parameter estimates from the heuristic estimation procedure are presented, inference is not the focus of this work and using the heuristic parameter estimation procedure for inference is not advised. Parameter estimates are presented to support the intuition motivating the modeling choices and provide relative comparisons of parameter estimates across states. The S1 Appendix provides a simulation study and discussion on the inferential limits of the heuristic parameter estimation procedure. Alternative heuristic estimation choices could be made and will be pointed out throughout the paper.

In this paper, s* will denote the flu season being forecasted. The past flu seasons (flu seasons occurring before season s*) used to estimate the parameters will be denoted with a subscript *s*. In practice and in this paper, when forecasting season s*, parameters are estimated from seasons s* − 1 and earlier. In what follows, I outline a six step procedure to estimate the unknown parameters *α*, ***γ***, $\sigma_{\mu }^{2}$, $\sigma_{\Sigma }^{2}$, λ, and *ϕ* and describe how to sample and forecast from Inferno’s posterior predictive distribution via MCMC.

#### 2.2.1 Step 1: Estimate *θ*_*s*,*t*_

The purpose of Step 1 is to estimate *θ*_*s*,*t*_. Estimating *θ*_*s*,*t*_ is not of value by itself, but is important as it facilitates the estimation of Inferno’s hyperparameters. The estimate of *θ*_*s*,*t*_, namely ${\hat{\theta }}_{s,t}$, is itself computed as a combination of two other quantities: ${\hat{\beta }}_{s,t}$ and ${\hat{\tau }}_{t}$. All computed quantities in Step 1 are based on training seasons only.

For a given geographic unit (e.g., state, HHS region, or the U.S.) and forecast season s*, let *y*_*s*,*t*_ be (w)ILI/100 for training season *s* ∈ 1, 2, …, *S* = s* − 1 and week of season *t*. First, compute ${\hat{\beta }}_{s,t}$ as a 3-week moving average:

$${\hat{\beta }}_{s,t=1}=\frac{1}{2}\left(y_{s,1}+y_{s,2}\right)$$ (7)

$${\hat{\beta }}_{s,1<t<T}=\frac{1}{3}\left(y_{s,t-1}+y_{s,t}+y_{s,t+1}\right)$$ (8)

$${\hat{\beta }}_{s,t=T}=\frac{1}{2}\left(y_{s,T-1}+y_{s,T}\right).$$ (9)

**Fig 1:**
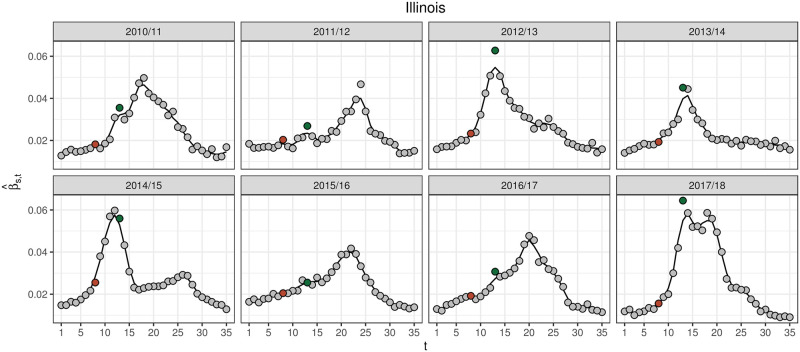
*y*_*s*,*t*_ (grey points) and ${\hat{\beta }}_{s,t}$ (black line) for the historical seasons for Illinois. *y*_*s*,*t*_ for the week of Thanksgiving (*t* = 8) and Christmas (*t* = 13) are highlighted in brown and green, respectively. ${\hat{\beta }}_{s,t}$ typically underestimates the sharp uptick in *y*_*s*,*t*_ observed on Christmas and to a lesser extent Thanksgiving, which is likely a result of changes in reporting and care-seeking behavior over the holidays.

Fig 1 shows the moving average fit to ILI/100 in Illinois. The purpose of ${\hat{\beta }}_{s,t}$ is to capture the time series trend in season *s* with a smooth, simple function that can be used to separate trend from noise in *y*_*s*,*t*_. By construction, the moving average captures the shape of the ILI/100 curve. Alternative smoothing functions, like smoothing splines [29], generalized ridge regression [30], or, with additional model assumptions, Kalman filtering [31] could also be used. The degree of smoothness in these alternative methods is controlled by a tuning parameter(s) and can be learned through cross-validation. I found a 3-week moving average worked well and, due to its simplicity, was appealing. The moving average, however, can miss sharp changes in *y*_*s*,*t*_ caused by differences in reporting practices over holidays. For instance, we see that the moving average most often underestimates *y*_*s*,*t*_ the week of Christmas (*t* = 13, or MMWR week 52).

To capture the systematic sharp changes in *y*_*s*,*t*_ that are common across training seasons, Inferno estimates the quantity *τ*_*t*_:

$${\hat{\tau }}_{t}=\frac{1}{S}\sum_{s=1}^{S}\left(y_{s,t}-{\hat{\beta }}_{s,t}\right).$$ (10)

**Fig 2:**
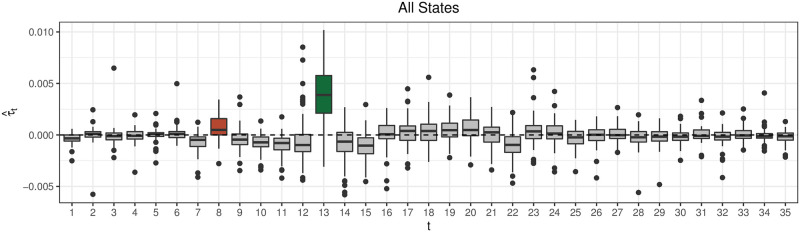
Each boxplot summarizes the quantity ${\hat{\tau }}_{t}$ across all states for each week *t*. ${\hat{\tau }}_{t}$ the week of Thanksgiving (brown) and Christmas (green) are systematically positive, likely as a result of systematic changes to reporting and care-seeking behavior over the holidays.

Fig 2 plots ${\hat{\tau }}_{t}$ for all states. ${\hat{\tau }}_{t}$ captures the holiday effects in *y*_*s*,*t*_, with a small but consistent positive ${\hat{\tau }}_{t}$ on the week of Thanksgiving (*t* = 8, or MMWR week 47) and a larger positive effect the week of Christmas.

Finally, the quantity ${\hat{\theta }}_{s,t}$ captures both the trend in *y*_*s*,*t*_ (${\hat{\beta }}_{s,t}$) and the holiday effects (${\hat{\tau }}_{t}$):

$$\begin{array}{cc} {\hat{\theta }}_{s,t} & =\{\begin{array}{cc} l & \text{if}\;{\hat{\beta }}_{s,t}+{\hat{\tau }}_{t}<l\\ 1-l & \text{if}\;{\hat{\beta }}_{s,t}+{\hat{\tau }}_{t}>1-l\\ {\hat{\beta }}_{s,t}+{\hat{\tau }}_{t} & \text{otherwise.} \end{array} \end{array}$$ (11)

where, again, *l* is a small number (in this paper, *l* = 0.0005) to ensure $0<{\hat{\theta }}_{s,t}<1$.

**Fig 3:**
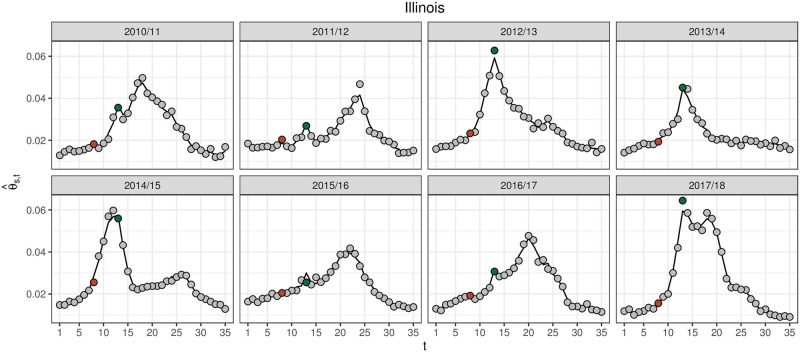
*y*_*s*,*t*_ (grey points) and ${\hat{\theta }}_{s,t}$ (black line) for the historical seasons for Illinois. *y*_*s*,*t*_ for the week of Thanksgiving (*t* = 8) and Christmas (*t* = 13) are highlighted in brown and green, respectively. ${\hat{\theta }}_{s,t}$ better matches *y*_*s*,*t*_ data on the holidays than ${\hat{\beta }}_{s,t}$ (Fig 1) by accounting for the systematic reporting and care-seeking changes over the holidays, as captured by ${\hat{\tau }}_{t}$.

Fig 3 shows how ${\hat{\theta }}_{s,t}$ tracks the profile of *y*_*s*,*t*_ by season, like ${\hat{\beta }}_{s,t}$, but better tracks *y*_*s*,*t*_ on the holidays, especially Christmas.

#### 2.2.2 Step 2: Estimate *α*

Inferno computes ${\hat{\theta }}_{s,t}$ in order to facilitate the estimation of the other unknown quantities of Inferno’s generative model. The expectation and the variance of Inferno’s data model (Eq 1) are,

$$\text{E}(y_{s,t}|\theta_{s,t},\alpha )=\theta_{s,t}$$ (12)

$$\text{Var}(y_{s,t}|\theta_{s,t},\alpha )=\frac{\theta_{s,t}\left(1-\theta_{s,t}\right)}{1+\alpha }.$$ (13)

The parameter *α* controls the variance of the data model, capturing the week-to-week variability in the ILI data. The larger *α* is, the smaller the variance, reflecting less week-to-week noise in the ILI data. The smaller *α* is, the larger the variance, reflecting more week-to-week noise in the ILI data. *α* > 0 is estimated by maximizing the likelihood of Inferno’s data model (or, equivalently, minimizing the negative log likelihood):

$$\hat{\alpha }=\underset{\alpha }{\text{argmin}}\sum_{s=1}^{S}\sum_{t=1}^{T}-\text{log}(\text{Beta}\left(y_{s,t}|{\hat{\theta }}_{s,t},\alpha \right)),$$ (14)

where log(*x*) is the natural log of *x*,

$$\text{Beta}(y_{s,t}|{\hat{\theta }}_{s,t},\alpha )=\frac{y_{s,t}^{a-1}{\left(1-y_{s,t}\right)}^{b-1}}{\text{B}(a,b)}$$ (15)

$$\text{B}(a,b)=\frac{\Gamma (a)\Gamma (b)}{\Gamma (a+b)}$$ (16)

$$a=\alpha {\hat{\theta }}_{s,t}$$ (17)

$$b=\alpha (1-{\hat{\theta }}_{s,t}),$$ (18)

and Γ() is the gamma function.

**Fig 4:**
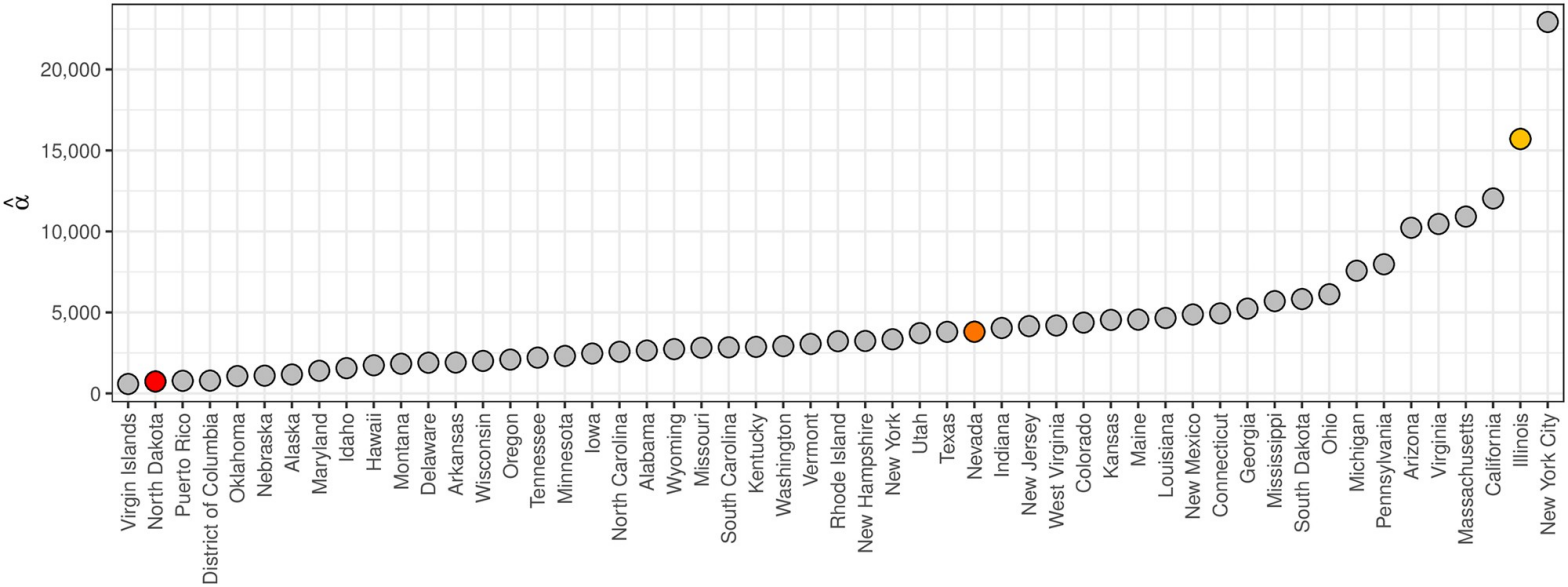
$\hat{\alpha }$ for all states based on training data from 2010/2011 through 2017/18. $\hat{\alpha }$ captures the week-to-week noise in ILI data that systematically varies from state-to-state, where North Dakota has more week-to-week noise than Illinois.

Fig 4 shows $\hat{\alpha }$ for all states, territories, and cities (collectively referred to as states). States like the U.S. Virgin Islands, North Dakota, and Puerto Rico have the smallest $\hat{\alpha }\text{s}$, reflecting they have the largest week-to-week noise in their ILI data, while states like California, Illinois, and New York City have the largest $\hat{\alpha }\text{s}$, reflecting they have the smallest week-to-week noise in their ILI data.

**Fig 5:**
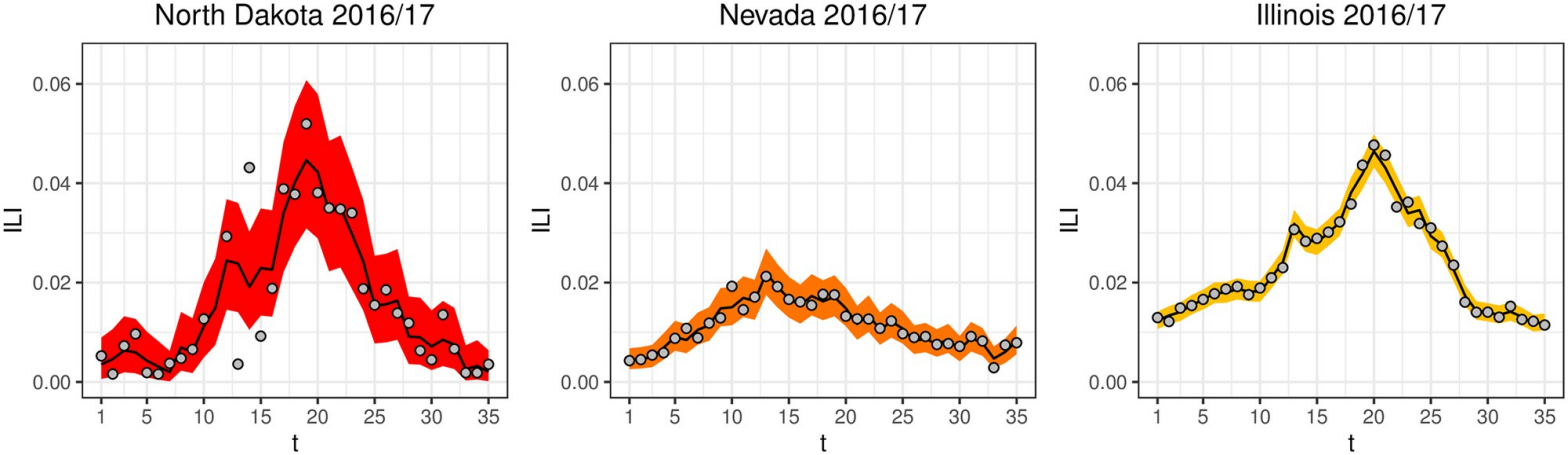
*y*_*s*,*t*_ (grey points), ${\hat{\theta }}_{s,t}$ (black line) and the 2.5 and 97.5 percentiles for the data model $\text{Beta}(\hat{\alpha }{\hat{\theta }}_{s,t},\hat{\alpha }\left(1-{\hat{\theta }}_{s,t}\right))$ (ribbon) for North Dakota, Nevada, and Illinois in 2016/17. $\hat{\alpha }$ captures the week-to-week noise in ILI data that systematically varies from state-to-state, where North Dakota has more week-to-week noise than Illinois.

Fig 5 shows summaries of the data model $\text{Beta}(\hat{\alpha }{\hat{\theta }}_{s,t},\hat{\alpha }\left(1-{\hat{\theta }}_{s,t}\right))$ for North Dakota, Nevada, and Illinois, illustrating the different levels of week-to-week noise in ILI data across states.

#### 2.2.3 Step 3: Estimate *γ*_*t*_

Seasonal flu has a typical shape to it in the United States. ILI starts at low levels early in the season, rises to a peak between December and March, and reverts to low levels by the end of May. The role of ***γ*** is to capture this typical seasonal flu profile. Inferno computes *γ*_*t*_ as follows:

$${\hat{\gamma }}_{t}=\frac{1}{S}\sum_{s=1}^{S}\text{logit}\left({\hat{\theta }}_{s,t}\right),$$ (19)

where logit(*p*) = log(*p*/(1 − *p*)).

**Fig 6:**
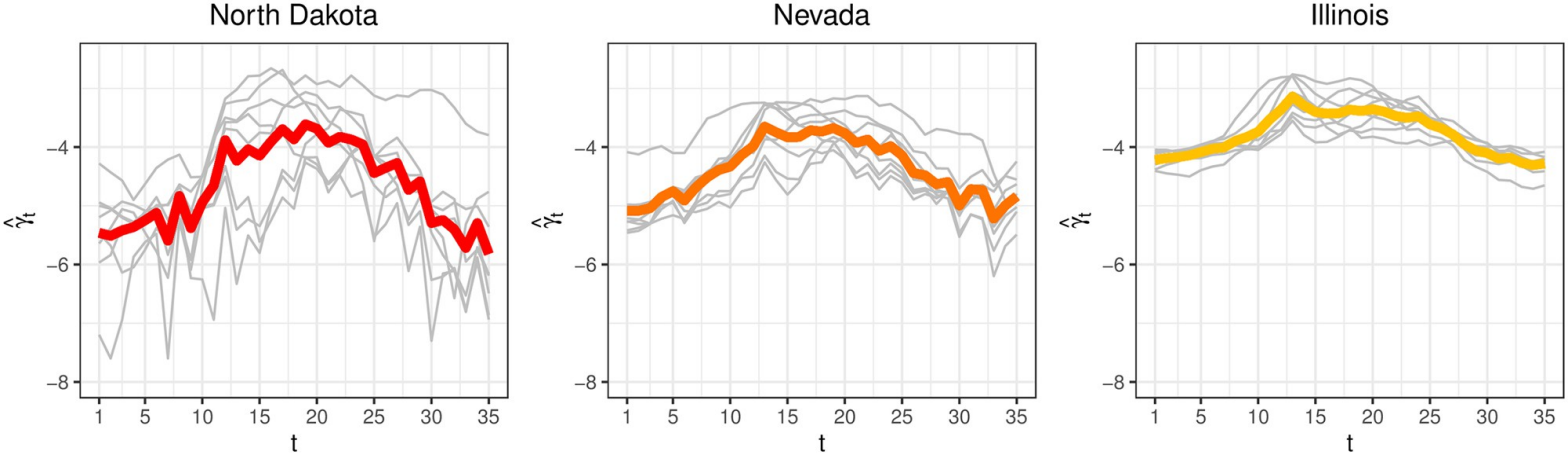
${\hat{\gamma }}_{t}$ (colored line) and $\text{logit}({\hat{\theta }}_{s,t})$ (grey lines) for North Dakota, Nevada, and Illinois. ${\hat{\gamma }}_{t}$ captures the typical profile of seasonal flu specific to each state on the logit scale.

Fig 6 shows $\hat{\gamma }$ for North Dakota, Nevada, and Illinois. We see for all states, $\hat{\gamma }$ captures the typical profile of seasonal flu on the logit scale, with low levels at the beginning of the flu season, ramping up to a peak in the middle, then reverting back to low levels by the end.

#### 2.2.4 Step 4: Estimate $\sigma_{\mu }^{2}$

Eq 2 is the mean of Inferno’s data model. While ***γ*** captures the typical profile of seasonal flu, ***δ***_*s*_ captures season-specific deviations from ***γ***. Inferno models ***δ***_*s*_ with a multivariate normal distribution (MVN):

$$\text{MVN}(\delta_{s}|\mu_{s},\Sigma )={\left(2\pi \right)}^{-T/2}{\left|\Sigma \right|}^{-1/2}\text{exp}(-\frac{1}{2}{\left(\delta_{s}-\mu_{s}1\right)}^{\prime }\Sigma^{-1}\left(\delta_{s}-\mu_{s}1\right)),$$ (20)

where **1** is a *T* × 1 vector of ones, **Σ** is a *T* × *T* positive semi-definite matrix, |**Σ**| is the determinant of **Σ**, and **Σ**^−1^ is the inverse of **Σ**. The model for the mean of the multivariate normal distribution, *μ*_*s*_, is

$$\mu_{s}∼\text{N}(0,\sigma_{\mu }^{2}).$$ (21)

Step 4 describes how to estimate $\sigma_{\mu }^{2}$.

First compute the following quantities:

$${\hat{\delta }}_{s,t}=\text{logit}\left({\hat{\theta }}_{s,t}\right)-{\hat{\gamma }}_{t}$$ (22)

$${\hat{\mu }}_{s}=\frac{1}{T}\sum_{t=1}^{T}{\hat{\delta }}_{s,t}.$$ (23)

**Fig 7:**
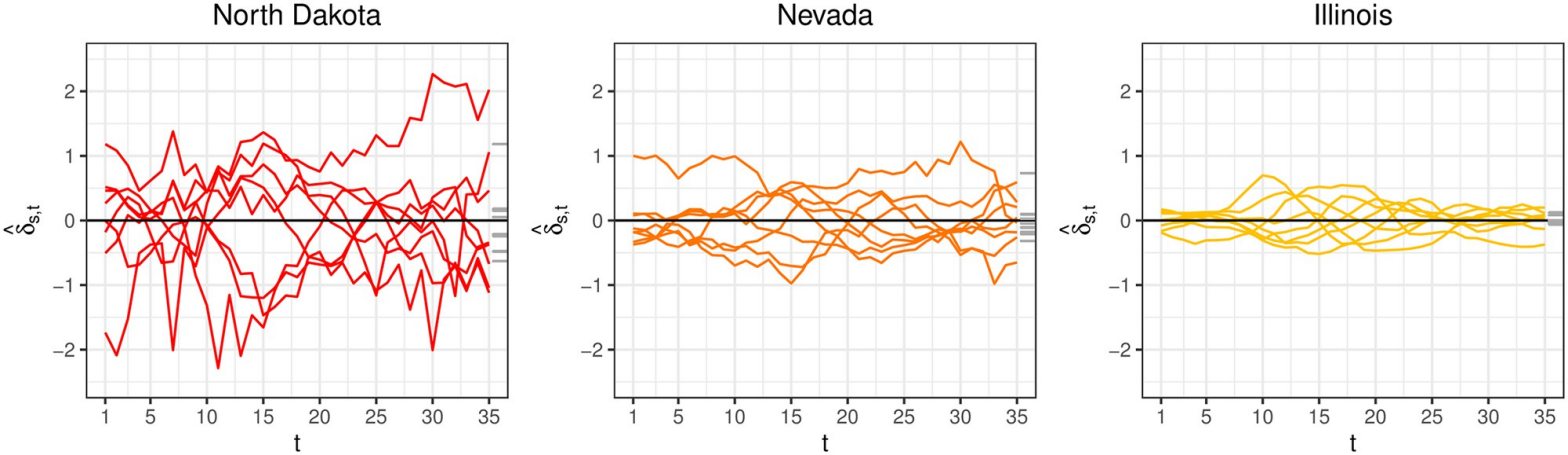
${\hat{\delta }}_{s}$ (colored lines) and ${\hat{\mu }}_{s}$ (grey tick marks) for North Dakota, Nevada, and Illinois. North Dakota exhibits more season-to-season variability in ${\hat{\mu }}_{s}$ than Illinois, as can be seen in the spread of ${\hat{\mu }}_{s}$.

By construction, $\sum_{s=1}^{S}{\hat{\delta }}_{s,t}=0$ for each *t*. Fig 7 shows ${\hat{\delta }}_{s}$ and ${\hat{\mu }}_{s}$ for North Dakota, Nevada and Illinois. The quantity ${\hat{\mu }}_{s}$ captures how far, on average, ${\hat{\delta }}_{s}$ deviates from **0**.

The quantity ${\hat{\sigma }}_{\mu }^{2}$ is computed as the unbiased sample variance:

$${\hat{\sigma }}_{\mu }^{2}=\frac{1}{S-1}\sum_{s=1}^{S}({\hat{\mu }}_{s}-\frac{1}{S}\sum_{s^{\prime }=1}^{S}{\hat{\mu }}_{s^{\prime }}{)}^{2}.$$ (24)

**Fig 8:**
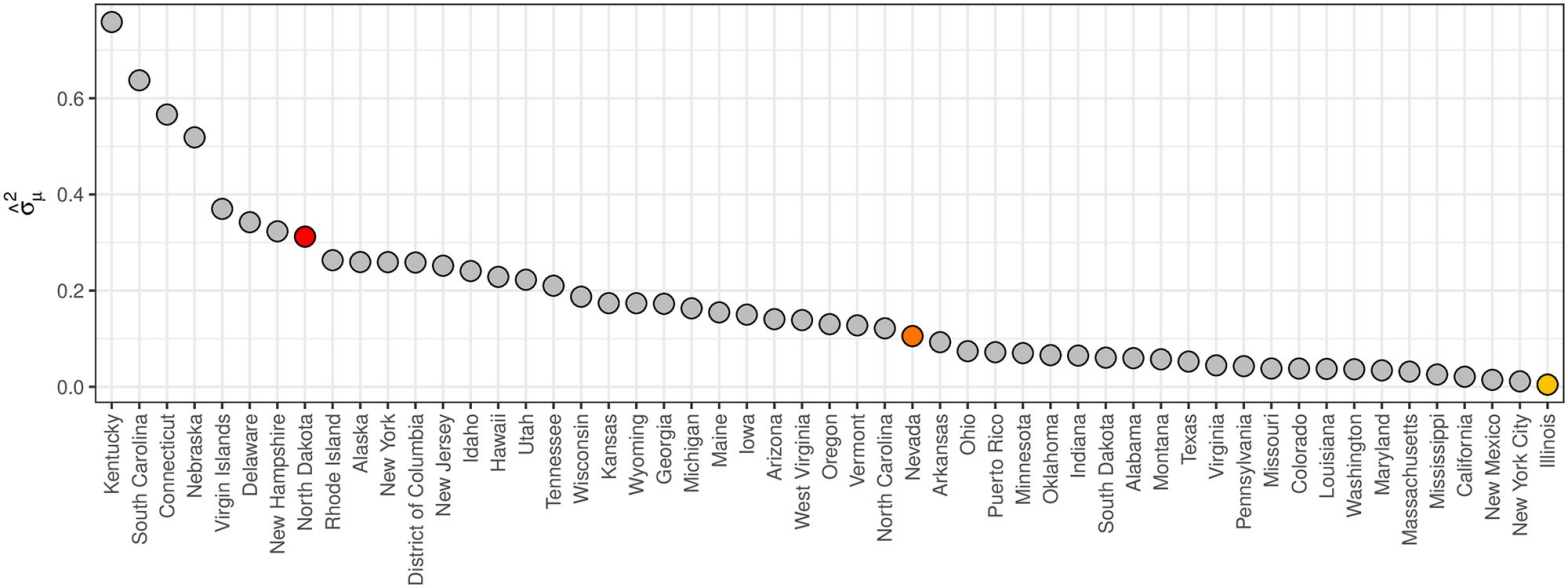
${\hat{\sigma }}_{\mu }^{2}$ for all states. Considerable variation in ${\hat{\sigma }}_{\mu }^{2}$ across states is observed.

Fig 8 shows ${\hat{\sigma }}_{\mu }^{2}$ for all states. Some states, like North Dakota, have appreciable average season-to-season variation while other states, like Illinois, have smaller average season-to-season deviations from their typical seasonal flu profiles.

#### 2.2.5 Step 5: Estimate $\sigma_{\Sigma }^{2}$, λ, *ϕ*

Step 5 estimates the covariance parameters in **Σ**. The covariance matrix captures different characteristics of ***δ***_*s*_. Recall Eqs 5 and 6:

$$\begin{array}{cc} \Sigma_{t,t} & =\sigma_{\Sigma }^{2}\\ \Sigma_{t,t^{\prime }\neq t} & =\phi \sigma_{\Sigma }^{2}\text{exp}\left(-\lambda {\left(t-t^{\prime }\right)}^{2}\right). \end{array}$$

Recall that *ϕ* ∈ [0, 1] and note that $\sigma_{\Sigma }^{2}$ can be written as $\phi \sigma_{\Sigma }^{2}+\left(1-\phi \right)\sigma_{\Sigma }^{2}$. By setting ${\sigma_{\Sigma }^{\prime }}^{2}=\phi \sigma_{\Sigma }^{2}$ and ${\sigma_{\epsilon }^{\prime }}^{2}=\left(1-\phi \right)\sigma_{\Sigma }^{2}$, Eqs 5 and 6 can be rewritten as:

$$\Sigma_{t,t}={\sigma_{\Sigma }^{\prime }}^{2}+{\sigma_{\epsilon }^{\prime }}^{2}$$ (25)

$$\Sigma_{t,t^{\prime }\neq t}={\sigma_{\Sigma }^{\prime }}^{2}\text{exp}\left(-\lambda {\left(t-t^{\prime }\right)}^{2}\right),$$ (26)

which is the standard parameterization of the squared exponential covariance function, where

- 1/λ is the correlation length scale parameter that determines the length of the wiggles of ***δ***_*s*_ (the larger 1/λ is, the longer the wiggles)
-
${\sigma_{\Sigma }^{\prime }}^{2}$ is the output variance that determines the amplitude of ***δ***_*s*_ (the larger ${\sigma_{\Sigma }^{\prime }}^{2}$ is, the larger the amplitude)
-
${\sigma_{\epsilon }^{\prime }}^{2}$ is an overdispersion parameter accounting for extra independent and identically distributed Gaussian noise added to the output variance (the smaller $\frac{{\sigma_{\epsilon }^{\prime }}^{2}}{{\sigma_{\Sigma }^{\prime }}^{2}}=\frac{\left(1-\phi \right)\sigma_{\Sigma }^{2}}{\phi \sigma_{\Sigma }^{2}}=\frac{1-\phi }{\phi }$ is, the smoother ***δ***_*s*_ is)

The marginal variance of ***δ***_*s*_ is $\sigma_{\Sigma }^{2}={\sigma_{\Sigma }^{\prime }}^{2}+{\sigma_{\epsilon }^{\prime }}^{2}$, the sum of the output variance and the overdispersion parameter. While the standard squared exponential parameterization of Eqs 25 and 26 are arguably more intuitive than the parameterization of Eqs 5 and 6, I found parameterizing ${\sigma_{\Sigma }^{\prime }}^{2}$ and ${\sigma_{\epsilon }^{\prime }}^{2}$ as $\phi \sigma_{\Sigma }^{2}$ and $\left(1-\phi \right)\sigma_{\Sigma }^{2}$, respectively, offered more numerical stability to the optimization described below as a result of *ϕ* being bounded between 0 and 1.

**Fig 9:**
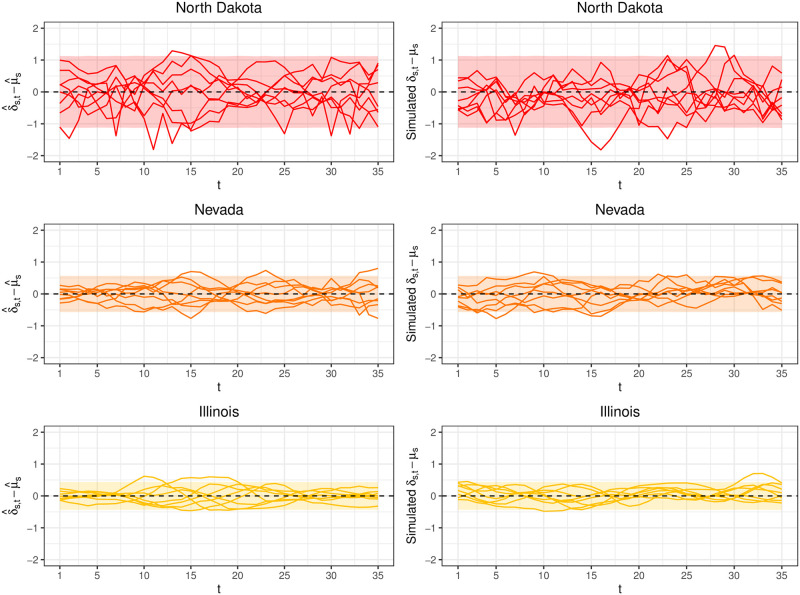
(Left column) The quantities ${\hat{\delta }}_{s}-{\hat{\mu }}_{s}1$ estimated from training data. Each line corresponds to a season *s*. The ribbon is the 95% confidence band from the fitted multivariate normal model. (Right column) The lines are realizations drawn from $\text{MVN}(0,\hat{\Sigma })$. The ribbon is the same 95% confidence band as in the left column for ease of comparison. Good visual agreement is seen between the simulated ***δ***_*s*_ − *μ*_*s*_**1** and ${\hat{\delta }}_{s}-{\hat{\mu }}_{s}1$ calculated from training data, suggesting the multivariate normal distribution is able to capture heterogenous discrepancy characteristics across states.

The left column of Fig 9 plots ${\hat{\delta }}_{s}-{\hat{\mu }}_{s}1$ for North Dakota, Nevada, and Illinois. North Dakota exhibits more variability than Illinois as can be seen with its wider range of values. Inferno estimates $\sigma_{\Sigma }^{2}$, a measure of how far ***δ***_*s*_ − *μ*_*s*_**1** typically deviates from **0**, as

$${\hat{\sigma }}_{\Sigma }^{2}=\frac{1}{ST-1}\sum_{s=1}^{S}\sum_{t=1}^{T}{\left({\hat{\delta }}_{s,t}-{\hat{\mu }}_{s}\right)}^{2}.$$ (27)

The remaining parameters of **Σ** are *ϕ* and λ. They collectively capture two different characteristics of ***δ***_*s*_. The parameter *ϕ* ∈ [0, 1] captures the roughness of ***δ***_*s*_. The larger (1 − *ϕ*)/*ϕ* is, the rougher ***δ***_*s*_ is. For instance, ${\hat{\delta }}_{s}$ for North Dakota in Fig 7 are much rougher than ${\hat{\delta }}_{s}$ for Illinois. The second characteristic of ***δ***_*s*_ captured by *ϕ* and λ is the correlation between entries of ***δ***_*s*_. The correlation between *δ*_*s*,*t*_ and *δ*_*s*,*t*+1_ is

$$\begin{array}{c} \text{Cor}\left(\delta_{s,t},\delta_{s,t+1}\right)=\frac{\text{Cov}(\delta_{s,t},\delta_{s,t+1})}{\sqrt{\text{Var}(\delta_{s,t})}\sqrt{\text{Var}(\delta_{s,t+1})}}=\frac{\phi \sigma_{\Sigma }^{2}\text{exp}\left(-\lambda {\left(t-\left(t+1\right)\right)}^{2}\right)}{\sigma_{\Sigma }^{2}}=\phi \text{exp}\left(-\lambda \right). \end{array}$$ (28)

Inferno estimates *ϕ* and λ by minimizing the negative log likelihood:

$$\begin{array}{cc} \hat{\lambda },\hat{\phi } & =\underset{\lambda ,\phi }{\text{argmin}}\sum_{s=1}^{S}-\text{log}(\text{MVN}({\hat{\delta }}_{s}|{\hat{\mu }}_{s},{\hat{\sigma }}_{\Sigma }^{2},\lambda ,\phi )). \end{array}$$ (29)

**Fig 10:**
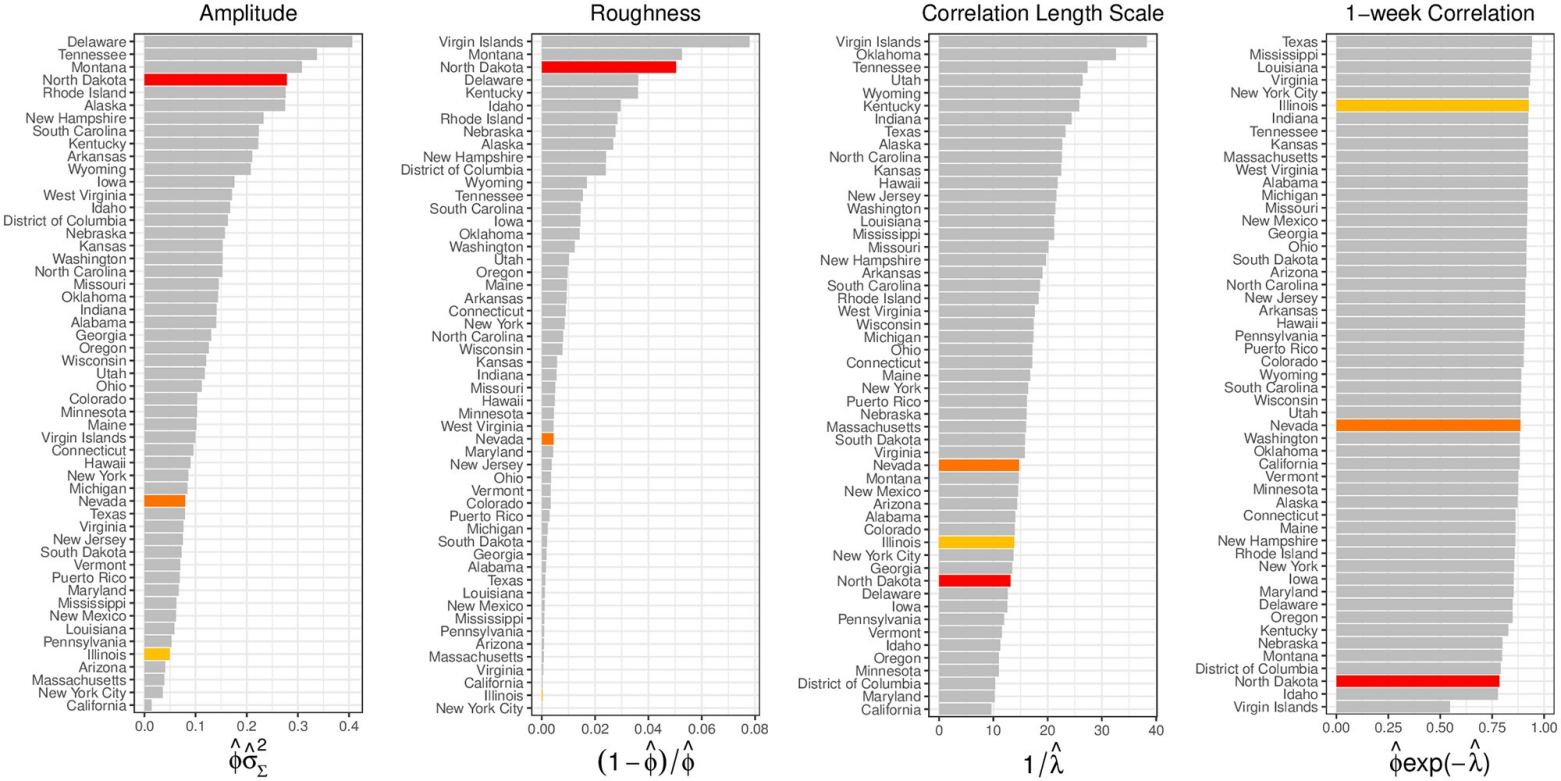
Functions of covariance parameter estimates for all states are presented for North Dakota, Nevada, and Illinois are highlighted in red, orange, and yellow, respectively. North Dakota has larger amplitude (larger $\hat{\phi }{\hat{\sigma }}_{\Sigma }^{2}$), is rougher (larger $\left(1-\hat{\phi }\right)/\hat{\phi }$), has a similar correlation length scale parameter estimate (similar $1/\hat{\lambda }$) and lower 1-week correlation ($\hat{\phi }\text{exp(}-\hat{\lambda })$ closer to 0) than Illinois.

Fig 10 plots functions of covariance parameter estimates for all states. Relative to Illinois, North Dakota has a larger amplitude (larger $\hat{\phi }{\hat{\sigma }}_{\Sigma }^{2}$), is rougher (larger $\left(1-\hat{\phi }\right)/\hat{\phi }$), has a similar correlation length (similar $1/\hat{\lambda })$ and has a lower 1-week correlation ($\hat{\phi }\text{exp}\left(-\hat{\lambda }\right)$ closer to 0).

The right column of Fig 9 shows realizations drawn from $\text{MVN}(0,\hat{\Sigma })$. The fitted multivariate normal distribution appears to do a good job capturing the different characteristics of the empirical quantities ${\hat{\delta }}_{s}-{\hat{\mu }}_{s}1$, suggesting the multivariate normal distribution is a defensible generative model for ***δ***_*s*_.

#### 2.2.6 Step 6: Sample forecasts from Inferno

The sixth and final step of Inferno is to replace parameters with their estimates and sample from the posterior predictive distribution. Recall s* is the forecast season and all parameters were estimated with data from seasons s* − 1 and earlier. Then, the generative model with parameters replaced by their estimates is

$$y_{s^{*},t}|\theta_{s^{*},t},\hat{\alpha }∼\text{Beta}(\hat{\alpha }\theta_{s^{*},t},\hat{\alpha }\left(1-\theta_{s^{*},t}\right))$$ (30)

$$\theta_{s^{*},t}={\text{logit}}^{-1}\left({\hat{\gamma }}_{t}+\delta_{s^{*},t}\right)$$ (31)

$$\delta_{s^{*}}|\mu_{s^{*}},\hat{\Sigma }∼\text{MVN}(\mu_{s^{*}}1,\hat{\Sigma })$$ (32)

$$\mu_{s^{*}}|{\hat{\sigma }}_{\mu }^{2}∼\text{N}(0,{\hat{\sigma }}_{\mu }^{2})$$ (33)

$${\hat{\Sigma }}_{t,t}={\hat{\sigma }}_{\Sigma }^{2}$$ (34)

$${\hat{\Sigma }}_{t,t^{\prime }\neq t}=\hat{\phi }{\hat{\sigma }}_{\Sigma }^{2}\text{exp}\left(-\hat{\lambda }{\left(t-t^{\prime }\right)}^{2}\right).$$ (35)

Given (w)ILI/100 observations for the first *t* weeks of flu season s* (i.e., given ***y***_s*,1:*t*_), Inferno forecasts the remainder of the flu season (weeks (*t* + 1) through *T*) by sampling from the posterior predictive distribution:

$$\begin{array}{cc} \left[{\tilde{y}}_{s^{*},\left(t+1\right):T}|y_{s^{*},1:t},\omega \right] & =\int \left[{\tilde{y}}_{s^{*},\left(t+1\right):T},\psi |y_{s^{*},1:t},\omega \right]d\psi =\int \left[{\tilde{y}}_{s^{*},\left(t+1\right):T}|\psi ,\omega \right]\left[\psi |y_{s^{*},1:t},\omega \right]d\psi , \end{array}$$ (36)

where [*X*|*Y*] is the conditional distribution of *X* given *Y* and ${\tilde{y}}_{s^{*},\left(t+1\right):T}$ is assumed to be independent of ***y***_s*,1:*t*_, given ***ψ*** and ***ω***, where ***ψ*** = {***θ***_s*,1:*T*_, ***δ***_s*,1:*T*_, *μ*_s*_} and ***ω*** = {$\hat{\alpha }$, $\hat{\gamma }$, ${\hat{\sigma }}_{\mu }^{2}$, ${\hat{\sigma }}_{\Sigma }^{2}$, $\hat{\lambda }$, $\hat{\phi }$}. The posterior predictive distribution of Eq 36 is not known in closed form. Markov chain Monte Carlo (MCMC) sampling is used to draw from the posterior predictive distribution. The probabilistic programming language JAGS (Just Another Gibbs Sampler) [32] is used to execute the MCMC sampling. JAGS is called with functions from the rjags package [33] in the programming language R [34]. The results are *J* draws from the posterior predictive distribution of Eq 36. For this paper, forecasts are based on *J* = 25, 000 samples, discarding the first 12,500 samples as burn-in and thinning the remaining 12,500 samples by two, resulting in forecasts based on 6,250 MCMC samples. A Markov chain should draw enough samples to achieve adequate estimation of the distribution(s) of interest. In general, when estimating quantiles of distributions, more samples are needed as the quantile of interest moves out into the tails of the distribution (i.e., it takes more samples to estimate the 99th percentile of a distribution well than it does to estimate the median of a distribution well). With more samples, however, comes increased runtime. I selected 25,000 samples as a practical balance between runtime and tail estimation quality. In practice, the amount of time available to run the MCMC will impact the number of samples a user selects. The JAGS code that implements Inferno can be found in the S1 Appendix.

**Fig 11:**
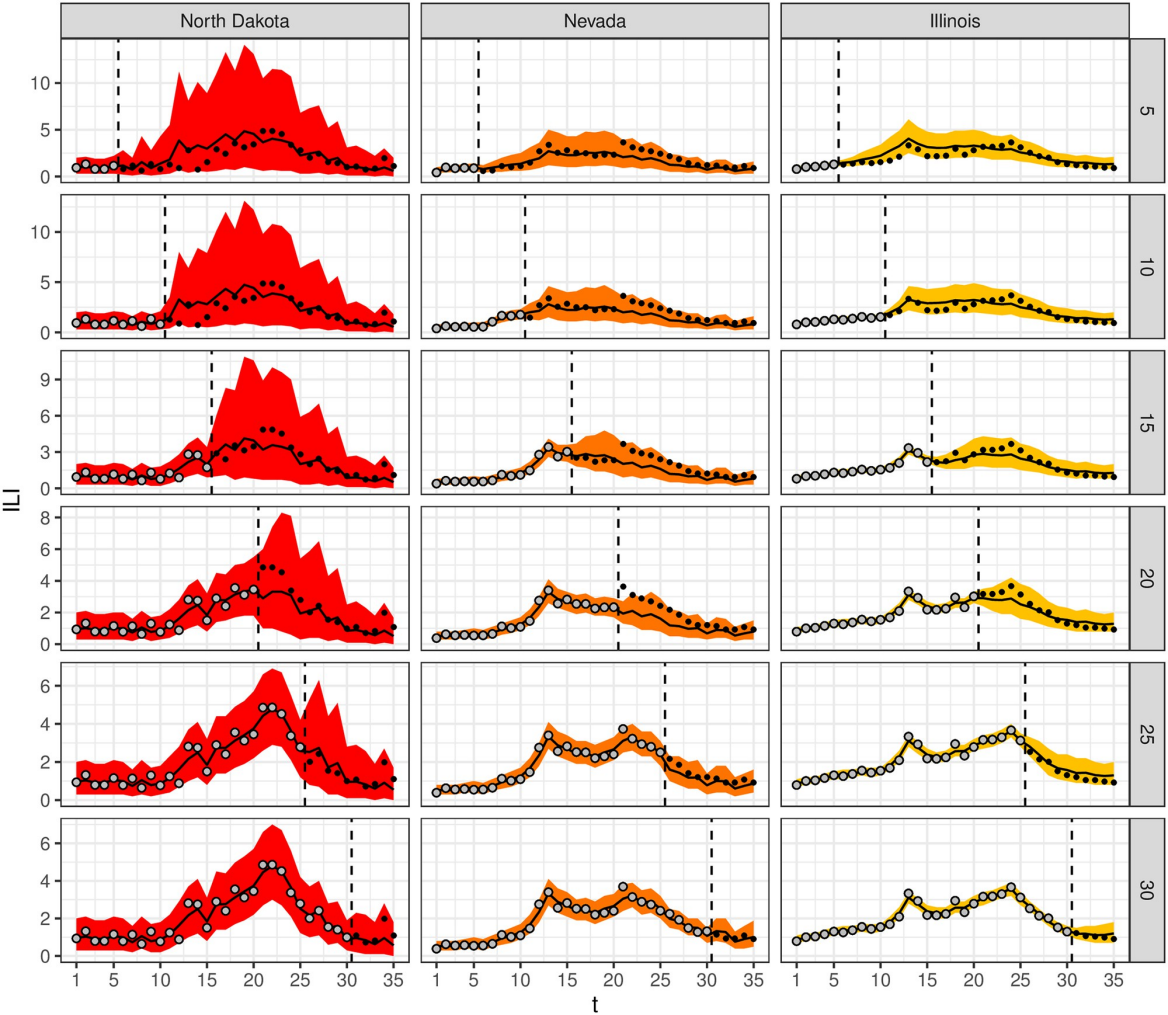
Inferno forecasts for the 2018/19 flu season for North Dakota, Nevada, and Illinois (columns) made *t* = 5, 10, 15, 20, 25, 30 weeks into the flu season based on summaries of draws from the posterior predictive distribution $\left[{\tilde{y}}_{s^{*},1:T}|y_{s^{*},1:t}\right]$ of Eq 36 (rows). Posterior mean (black line) and 95% prediction intervals (ribbons) are displayed, along with ***y***_s*,1:*t*_ (grey points) and ***y***_s*,(*t*+1):*T*_, the future (w)ILI/100 values being forecasted (black points). The ribbon for times 1 to *t* is a summary of the fit to data ***y***_s*,1:*t*_, while the ribbon for times *t* + 1 to *T* is a summary of the forecast for season s*.

Fig 11 shows the forecasts for North Dakota, Nevada, and Illinois throughout the 2018/19 flu season. The presented summaries of the forecasts are the posterior predictive means and the 95% posterior prediction intervals.

## 3 Results

To evaluate Inferno’s forecasting performance, Inferno is pseudoprospectively compared to all models that participated in the U.S. CDC’s 2018/19 National and Regional FluSight challenge as well as the State challenge. Forecasting follows the guidelines outlined by the CDC FluSight challenge; see [26] for details. The forecasts and the evaluation procedure is briefly described below.

Forecasts are made for four short-term targets (1, 2, 3, and 4-week-ahead) and three seasonal targets (the peak week, the peak percentage, and the onset week—onset is not forecasted for the State challenge). All forecast targets are binned and a probability is assigned to each bin such that the sum of all probabilities over all bins for a target equals 1. The bins for the onset week and the peak week are bins of one week; the bins for the short-term targets and the peak percentage are tenths of a percent (e.g., a bin from 2.0% (included) to 2.1% (excluded)) from 0 to 13%, with one large bin from 13% to 100%.

Define bin *b* as the bin containing the correct target, *B* as the set of all bins that will be scored (where *b* ∈ *B*), and *p*_*B*_ ∈ [0, 1] as the sum of the probabilities assigned to all the bins in *B*. The *modified log score* used by FluSight is computed as max(−10, log(*p*_*B*_)). When *B* = *b*, the modified log score is called the *single-bin log score* and is the scoring criteria used starting with the 2019/20 FluSight challenge. When *b* ∈ *B* but *b* ≠ *B*, the log score is called the *multi-bin log score* and was the scoring criteria used in the 2018/19 FluSight challenge. The multi-bin log score essentially scores the forecast probability assigned to not only the correct target bin, but also all target bins that are “close” to the correct target bin. The change from multi-bin log score to single-bin log score is motivated by the topic of proper/improper scoring rules [35]. For a recent, detailed discussion on this, the interested reader is directed to [24] and [25]. Finally, multi-bin skill and single-bin skill are derived by exponentiating the multi-bin and single-bin log scores, respectively. Single- and multi-bin skill are ∈ (0, 1], with larger skills being better.

The (w)ILI data are subject to weekly revisions. As a result, it is important to use the (w)ILI estimates that were available at the time to make faithful comparisons to models that participated in the real-time FluSight challenges. Data available on historical dates are made available by the Carnegie Mellon University Delphi group’s API [36] and were used to produce the results of the pseudoprospective comparison to real-time FluSight participating models.

**Fig 12:**
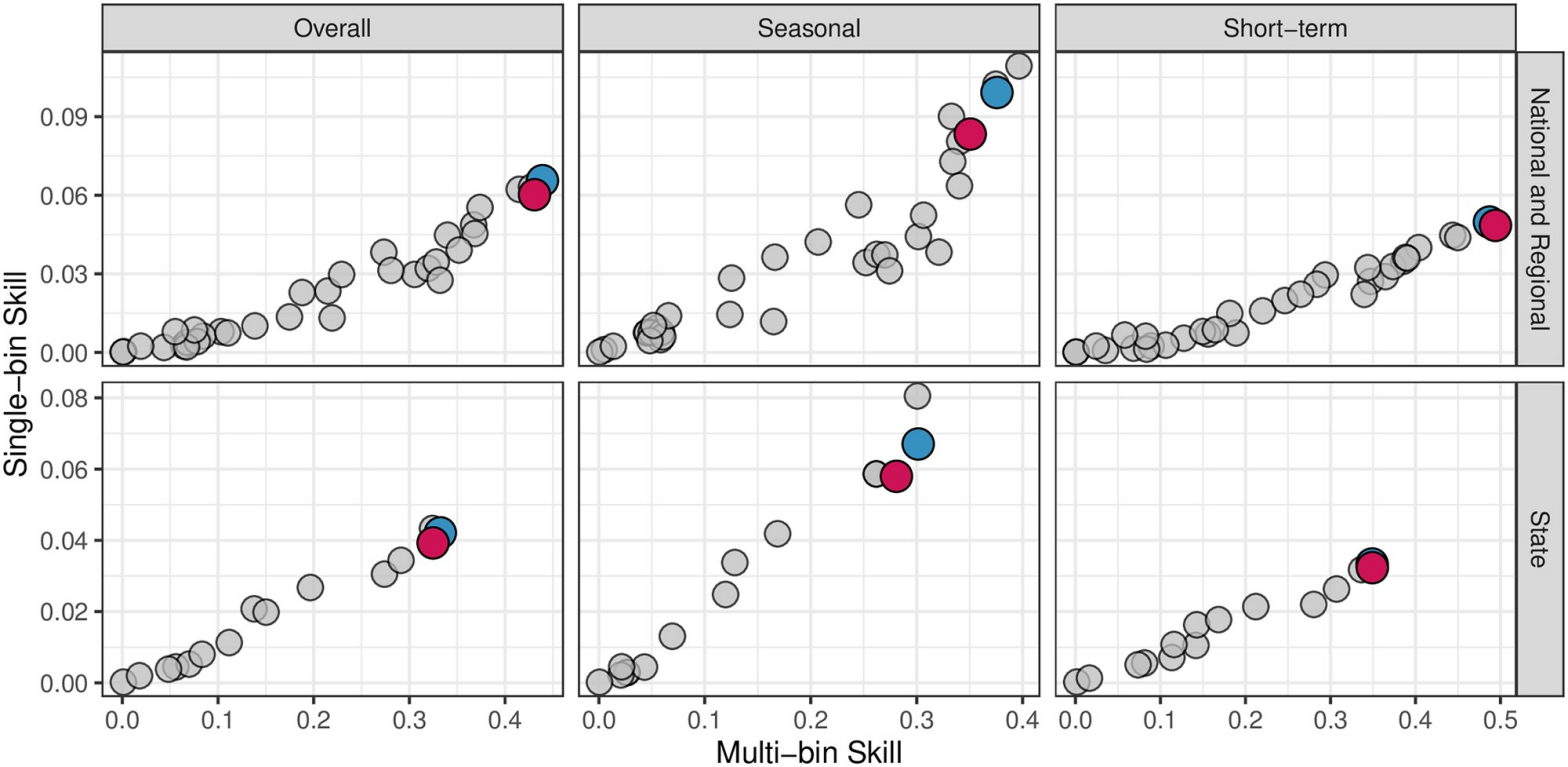
Results for the 2018/19 FluSight National and Regional challenge (top row) and State challenge (bottom row) for Inferno (red point), Dante (blue point) and all other models that participated in the 2018/19 FluSight challenges (grey points). The 2018/19 FluSight challenge evaluated models using multi-bin skill (x-axis), but starting with the FluSight 2019/20 challenge, will be using single-bin skill (y-axis). Skill scores are presented overall (left column), but also by seasonal targets (middle column) and short-term targets (right column). Inferno is a leading forecasting model overall, excelling in short-term forecasting, with good but not leading seasonal forecasting performance.

Fig 12 and Table 1 show the multi- and single-bin skills for Inferno and all models that participated in the 2018/19 FluSight challenges. Inferno would have placed 2nd only to Dante in the 2018/19 FluSight National and Regional as well as State challenges. FluSight 2018/19 used multi-bin skill as the forecast evaluation metric. Starting with FluSight 2019/20, single-bin skill will be used. While single-bin and multi-bin skills are correlated, as can be seen in Fig 12, the relationship is not perfect. Models can rise or fall in the relative ranking depending on which evaluation metric is used for scoring, highlighting that the evaluation metric the forecasting challenge organizing body selects is of consequence. Inferno and Dante both perform better under the multi-bin skill evaluation than single-bin skill, but are both top 4 models by either evaluation metric. Most importantly, the drop in predictive performance from Dante to Inferno is small.

**Table 1:** The rank by challenge and target for Inferno and Dante as measured by single-bin and multi-bin skill. Inferno would have placed 2nd in both the National and Regional and the State challenges as measured by multi-bin skill, only finishing behind Dante. Inferno would have placed 4th (National and Regional) and 3rd (State) were the forecasts evaluated with single-bin skill. For both challenges and both evaluation metrics, Inferno achieved better short-term than seasonal performance.

2018/19 FluSight Challenge	Target	Multi-bin Rank		Single-bin Rank	
		Inferno	Dante	Inferno	Dante
**National and Regional** **(34 models)**	**Overall**	2	1	4	1
	**1 wk ahead**	1	2	1	2
	**2 wk ahead**	1	2	2	1
	**3 wk ahead**	1	2	2	1
	**4 wk ahead**	2	1	2	1
	**Season peak percentage**	5	1	5	3
	**Season peak week**	11	8	11	8
	**Season onset**	5	1	7	1
**State** **(15 models)**	**Overall**	2	1	3	2
	**1 wk ahead**	3	1	3	1
	**2 wk ahead**	2	1	2	1
	**3 wk ahead**	1	2	2	1
	**4 wk ahead**	1	2	1	2
	**Season peak percentage**	3	2	5	2
	**Season peak week**	3	1	3	1

**Fig 13:**
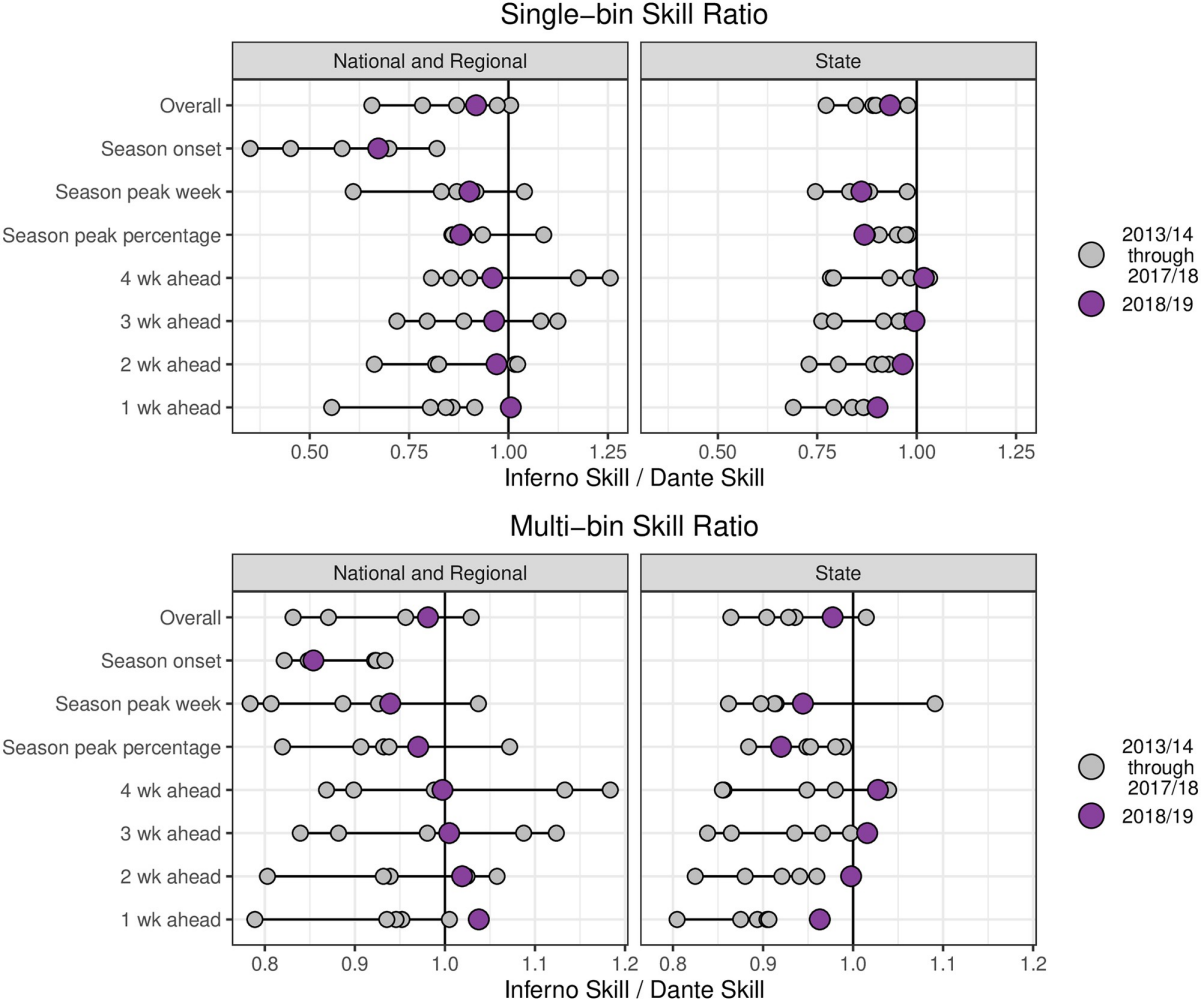
Retrospective comparison of Dante’s and Inferno’s single-bin (top) and multi-bin (bottom) skills for the 2013/14 through 2017/18 flu seasons (grey points) and the pseudoprospective comparison of the 2018/19 season (purple points). Skill ratios less than one (Inferno/Dante) indicates better performance by Dante. For most season/targets, Dante had a higher skill than Inferno. Inferno’s relative performance to Dante in 2018/19 was largely consistent with past season comparisons, with Inferno’s short-term forecasts for states performing better than usual in 2018/19 relative to Dante than in past years.

The small drop in performance from Dante to Inferno in 2018/19 is largely consistent with other seasons. Fig 13 shows Inferno’s skill relative to Dante’s skill when retrospectively compared for seasons 2013/14 through 2017/18 (using data from MMWR week 40 of 2010 through the forecast data for training/fitting). For the majority of seasons and targets, Inferno’s performance is worse than Dante’s by a small margin. From Fig 13, we can see that, relative to Dante, Inferno performed better than expected in 2018/19 for short-term targets at the state level. For all other regions and targets, however, Inferno’s drop in performance relative to Dante in 2018/19 is consistent with the drop in performance seen in other seasons, suggesting the relatively small drop in performance for Inferno is typical. For context, Inferno’s average overall multi-bin skill was 94% of Dante’s overall multi-bin skill for the National and Regional challenge. If each model that participated in the 2016/17, 2017/18, or 2018/19 National and Regional FluSight challenge had its overall multi-bin skill reduced by 6%, the average drop in rank was just over 1 position (i.e., if a model finished in Xth place in the challenge, a 6% reduction in its skill would, on average, result in that same model finishing in (X+1)th place). The drop in rank increases from 1 position to almost 3 positions if you focus only on the models that finished in the top 10, indicating that a 6% drop in skill has a greater impact on a model’s relative rank for better performing models than worse performing models. The retrospective comparison shown in Fig 13 ignores revisions made to data in real-time (i.e., the validation data is used for forecasting as the data that would have been available in real-time is not available back to 2013/14). As a result, Inferno’s and Dante’s forecasts are comparable to each other but not real-time forecasts.

**Fig 14:**
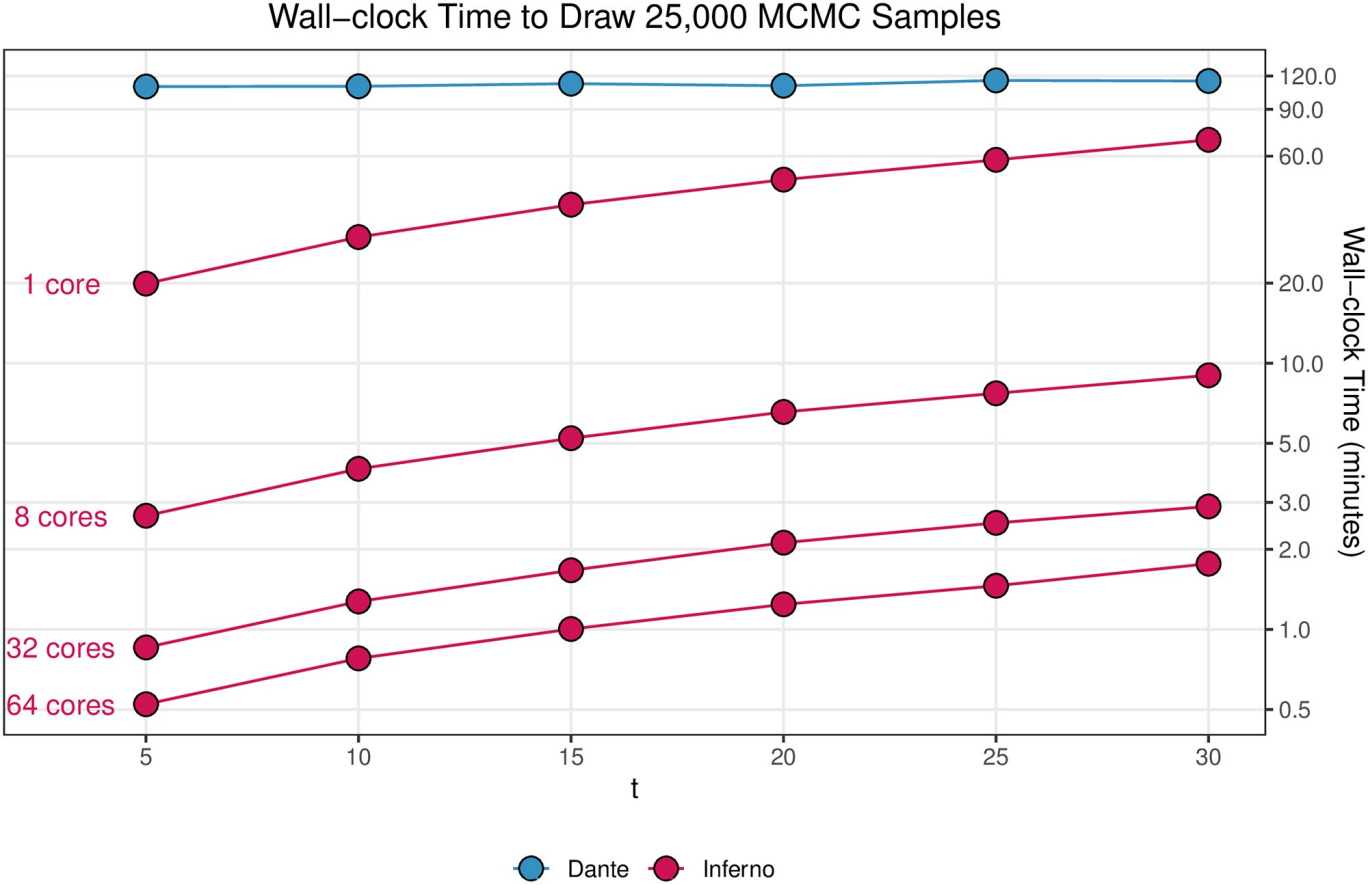
The wall-clock runtime of Inferno (red) and Dante (blue) to draw 25,000 MCMC samples for all 64 geographies. The red lines correspond to the runtime of Inferno based on 1, 8, 32, or 64 computing cores. Total runtime decreases as the number of cores increases. Total runtime increases as the size of the conditioning data increases for both Dante and Inferno. Dante draws 25,000 MCMC samples for all 64 geographies in 110 to 120 minutes. Dante’s reported runtimes are not monotonically increasing due to noise, as only one run was performed at each *t*. With 64 cores, it takes Inferno between 30 seconds and 2 minutes to draw 25,000 MCMC samples for all 64 geographies. When all 64 geographies of Inferno are run serially (1 core), Inferno takes between 20 and 70 minutes.

The small drop in predictive performance from Dante to Inferno is offset by Inferno’s significant improvement in runtime for real-time forecasting and preparation for future scalability to more granular forecasting geographies (e.g., county-level). Fig 14 shows the runtime comparison between Dante and Inferno at different stages of the flu season and number of cores to draw 25,000 MCMC samples for all 64 geographies (53 states, 10 HHS regions, and the United States). Dante takes between 110 and 120 minutes, while Inferno takes between 20 and 70 minutes (if run serially on one core). Inferno, however, can be trivially parallelized for real-time forecasting. As a result, Inferno can draw the same 25,000 MCMC samples for all geographies in 30 seconds to 2 minutes when fully parallelized (running one geography per core). Fig 14 shows that Inferno improves runtime relative to Dante in two ways: by being a simpler model with fewer parameters and latent quantities to sample (comparing Dante to 1 core Inferno runtimes) and by being parallelizable (comparing Dante to 8, 32, and 64 core runtimes).

## 4 Discussion

In this paper, I argued that while predictive performance is the most important measure of a forecasting model, other factors like runtime are important for model development, scalability, and meeting real-time, operational timelines. Developing a model with leading predictive performance but drastically improved runtime was the motivation behind Inferno. I laid out a six step procedure to heuristically estimate the parameters of Inferno from historical (w)ILI data, greatly reducing the MCMC computations as executed by the probabilistic programming language JAGS. Furthermore, by forecasting each geography separately, Inferno can take advantage of parallelization, both improving forecast runtimes in the present while being scalable and well-positioned for the more spatially granular future of flu forecasting (e.g., county-level forecasting).

Inferno’s predictive performance was comparable to but worse than Dante’s. This may be for a couple different reasons, both of which are addressable. Firstly, Dante explicitly models revisions; previous work has shown that accounting for and modeling revisions can result in improved predictive performance [16, 20]. Similar modeling can be incorporated into Inferno at little additional computational cost. Secondly, Dante models correlation across states within a season by coupling states within a hierarchical framework. This coupling comes at a computational cost. The price Inferno pays to achieve significant computational speed-ups is the loss of coupling. There has been some recent work that takes independently generated probabilistic forecasts and, using principles of coherence, produces self-consistent forecasts that have improved predictive performance [37–39]. The goal of this two staged approach is to achieve the computational speed ups parallelization offers to independently generated forecasts and then, through post-hoc coupling (i.e., coherence), recover some of the lost forecast performance. The combination of revision modeling and coherence exploitation may result in equal or even better predictive performance at minimal computational cost.

In this paper, I discussed the importance of forecasting challenges to help direct forecasting model development. Forecasting models are tools to help us answer questions. Forecasting challenges articulate what questions we want to answer and help define what properties we want our forecasting tools to have. They do this by selecting data sources, targets, scoring rules, geographic scope, and timelines that incentivize the development of models to optimize a forecast score while meeting these operational constraints. With the recently announced U.S. CDC Center for Forecasting and Outbreak Analytics [40], there are exciting opportunities for the growth and influence of forecasting challenges to flourish.

## Supporting information

S1 Appendix The JAGS code implementing Inferno’s MCMC sampling routine is found in Section 1. A simulation study illustrating the inferential limits of Inferno’s heuristic parameter estimation procedure is found in Section 2.
(PDF)
