# Supplementary material for: Fast and accurate influenza forecasting in the United States with Inferno
Source: PLoS Comput Biol. 2022 Jan 31;18(1):e1008651. doi: 10.1371/journal.pcbi.1008651 (PMC8830797; doi:10.1371/journal.pcbi.1008651)
Supplement: S1 Appendix — A simulation study illustrating the inferential limits of Inferno’s heuristic parameter estimation procedure is found in Section 2. (PDF) [file pcbi.1008651.s001.pdf]

Supporting Information: Fast and Accurate Influenza Forecasting in the United States with Inferno

Dave Osthus

Statistical Sciences Group, Los Alamos National Laboratory, Los Alamos, New Mexico, United States of America

Inferno JAGS Code

The following inputs are supplied to the JAGS model that implements Inferno:

- `T` is 35, the number of weeks of the flu season.
- `y` is a 35×1 vector where `y[t]` is the observed (w)ILI/100 value for week `t` if it has been observed or `NA` if it has not. If `y[t]` is less than 0.0005 or greater than 0.9995, `y[t]` is set equal to 0.0005 or 0.9995, respectively.
- `alpha` is $\hat{\alpha}$, computed from Equation 14Step 2: Estimate αequation.2.14.
- `gamma` is a 35×1 vector where `gamma[t]` is $\hat{\gamma}_t$, computed from Equation 19Step 3: Estimate $\gamma_t$equation.2.19.
- `invCholUpper` is the inverse of the upper triangular Cholesky decomposition of $\hat{\mathbf{\Sigma}}^{-1}$, where $\hat{\mathbf{\Sigma}}$ is computed from Equation 34Step 6: Sample Forecasts from Infernoequation.2.34 and 35Step 6: Sample Forecasts from Infernoequation.2.35.
- `sigma_mu` is the square root of $\hat{\sigma}^2_{\mu}$, computed from Equation 24Step 4: Estimate σ^2_{μ}equation.2.24.

The JAGS code implementing Inferno is as follows:

```
model{
  for(t in 1:T){
    ## draw from posterior predictive distribution
    ypred[t] ~ dbeta(alpha*theta[t], alpha*(1-theta[t]))
    ## data model
    y[t] ~ dbeta(alpha*theta[t], alpha*(1-theta[t]))
    ## compute theta
    theta[t] <- ilogit(gamma[t] + delta[t])
  }
  ## discrepancy GP model
  delta[1:T] <- mu + invCholUpper %*% Z[1:T]
  ## sample standard normals
  for(t in 1:T){
    Z[t] ~ dnorm(0,1)
  }
  ## discrepancy mean model
  mu ~ dnorm(0,pow(sigma_mu,-2))
}
```

Simulation Study

In this simulation study, I investigate the ability of the heuristic parameter estimation procedure of Section 2.2 to recover the parameters used to simulate mock data.

For each state, I take $\hat{\alpha}$, $\hat{\phi}$, $\hat{\lambda}$, $\hat{\sigma}^2_\Sigma$, and $\hat{\sigma}^2_\mu$ from the heuristic estimation procedure presented in Figs 4, 5, and 10 and simulate $y_{s,t}$ 500 times eight seasons for each state. For each of the 500 sets of eight simulated seasons, I run Inferno's heuristic parameter estimation procedure, resulting in estimates of $\hat{\alpha}$, $\hat{\phi}$, $\hat{\lambda}$, $\hat{\sigma}^2_\Sigma$, and $\hat{\sigma}^2_\mu$. Fig S1 presents the central 95% of the 500 parameter estimates (grey lines) and the parameter value used to generate the data (points). Red points represent parameter values that fell outside the 95% interval; grey points fell within.

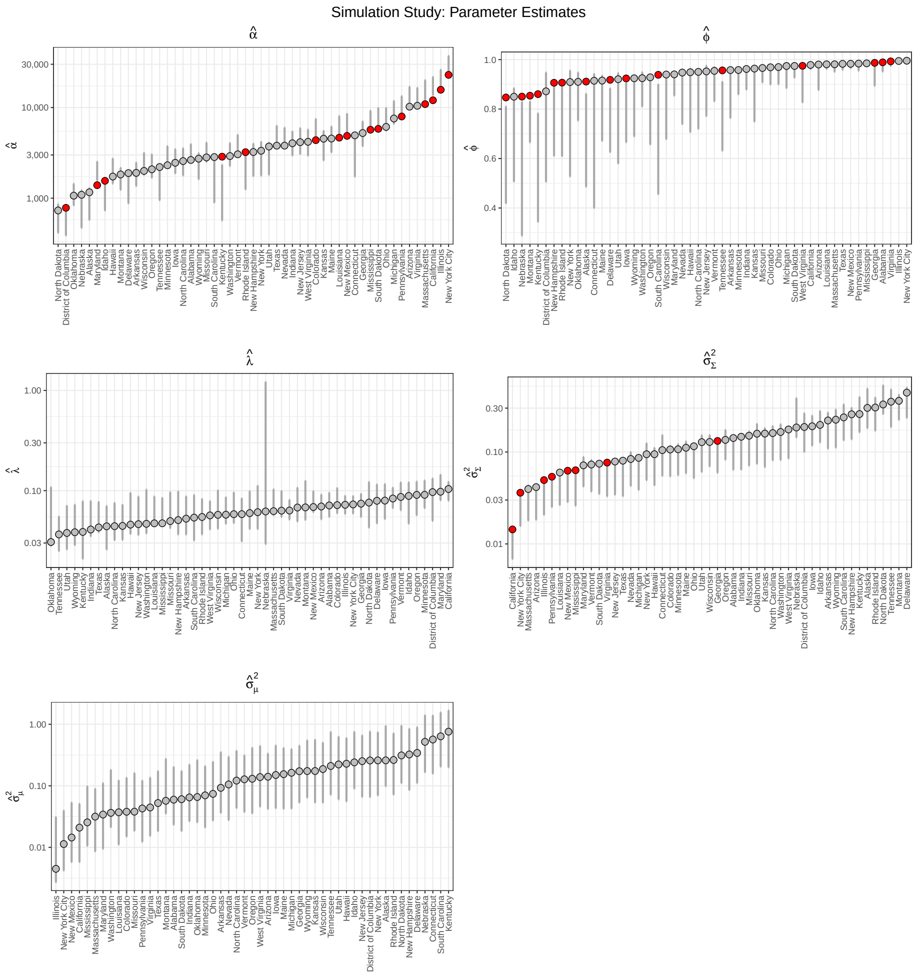

Fig S1. Parameter estimates from the simulation study. Grey lines denote the 2.5th and 97.5th percentiles of the 500 estimated parameters. Points are the parameter values used to simulate the data. Grey points are generating parameter values within the 2.5th and 97.5th percentiles; red points fall outside.

As can be seen, the heuristic parameter estimation procedure is able to recover λ and σ_μ^2 well, it recovers σ_Σ^2 for values over 0.1 well but tends to underestimate σ_Σ^2 for values less than 0.1, it tends to underestimate ϕ, and overestimate large values of α.

As mentioned in Section 2.2, it is not recommended to use the heuristic parameter estimation procedure if inference is of interest. A full maximum

likelihood estimation approach, or, with additional modeling assumptions, a full Bayesian fitting should be considered. However, the purpose for presenting parameter estimates in the Section 2.2 was to provide modeling intuition by comparing state parameter estimates in a relative sense. Fig S2 shows the actual versus the estimated rank of the parameters, using the median estimated parameter of the 500 estimates (rank 1 means the smallest parameter value). We see from Fig S2 that the heuristic parameter estimation procedure does do a good job estimating the relative parameter rank, indicating that while the parameter estimates themselves may be biased (for some parameters), the relative comparison is useful.

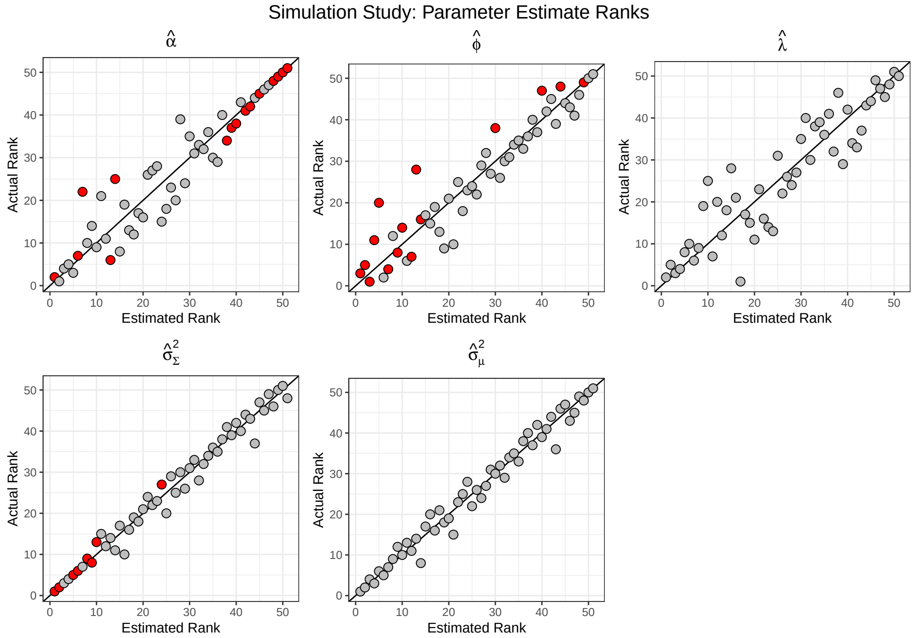

Fig S2. The actual parameter rank (y-axis) versus the estimated parameter rank (x-axis), where rank of 1 means the smallest estimate. Grey points correspond to generating parameter values that fell within the 2.5th and 97.5th percentiles of the 500 estimated parameters; red points fall outside. While the heuristic parameter estimation procedure struggles to estimate α, ϕ, and low values of σ^2_Σ, it does a reasonable job estimating the rank of the parameters.
